# Supplementary material for: The molecular and functional landscape of resistance to immune checkpoint blockade in melanoma
Source: Nat Commun. 2023 Mar 18;14:1516. doi: 10.1038/s41467-023-36979-y (PMC10024679; doi:10.1038/s41467-023-36979-y)
Supplement: Supplementary file 1 — Supplementary Information [file 41467_2023_36979_MOESM1_ESM.pdf]

Figure S1

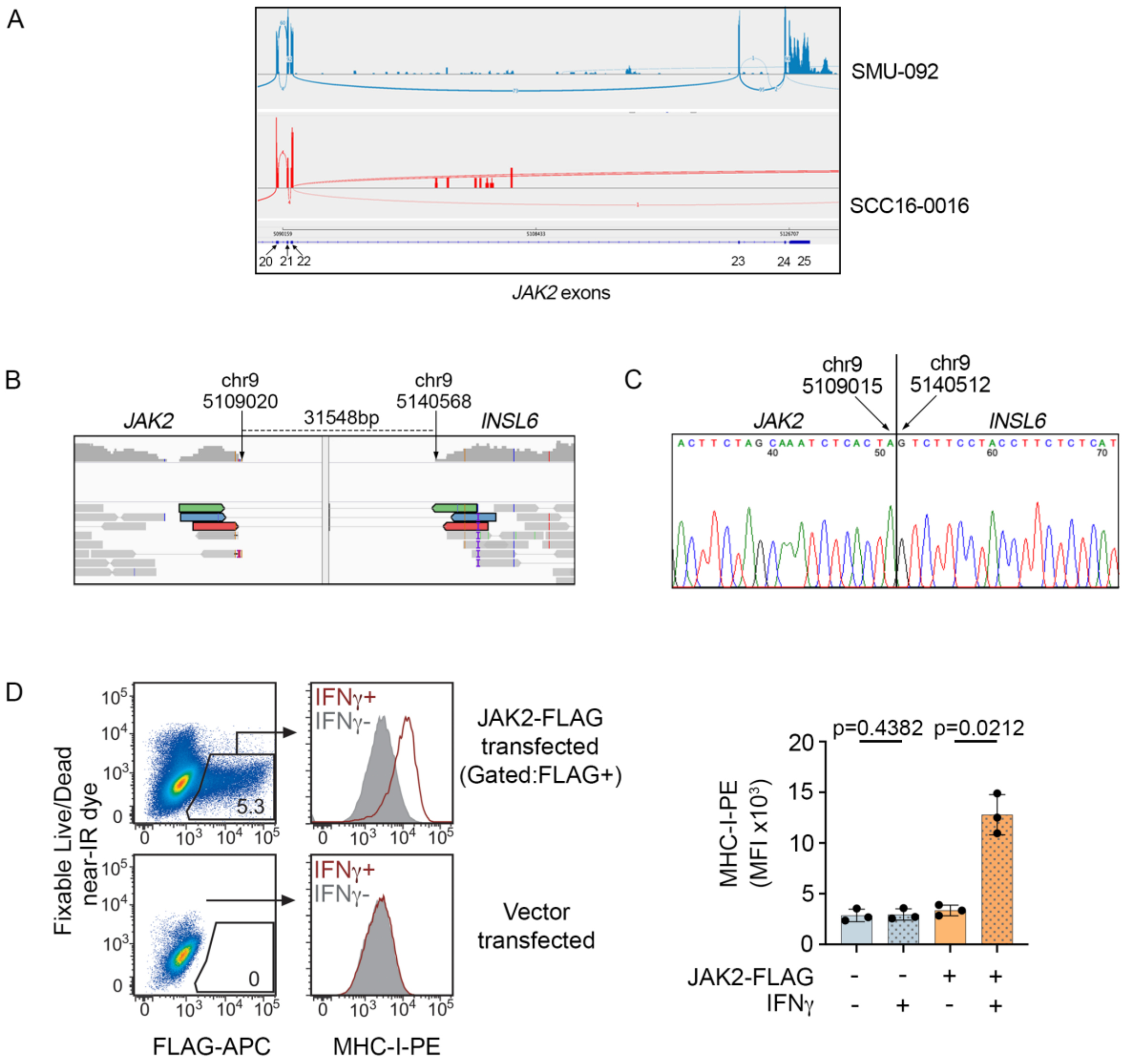

Fig. S1: Whole exome and genome analysis and functional validation of SCC16-0016

A. Transcriptome analysis of the SCC16-0016 PD1 PROG cell line (not responsive to IFNγ stimulation) showing *JAK2* deletion compared to SMU-092 (responsive to IFNγ stimulation). *JAK2* exons 20-25 are shown.

B. Whole genome sequencing analysis of the SCC16-0016 PD1 PROG cell line showing *JAK2* deletion. Chromosome positions are based on human genome assembly hg38.

C. Chromatogram from Sanger sequencing of the SCC16-0016 PD1 PROG cell line DNA showing fusion between the chromosome 9p24.1 genes *JAK2* and *INSL6.*

D. Scatter plots showing expression of MHC-I on JAK2-FLAG-transfected (expression shown for FLAG+ cells) and vector control-transfected SCC16-0016 cells, following 48h treatment with IFNγ (1000 U/ml). Histogram shows the mean±sd of MHC-I-PE level (median fluorescence intensity; MFI, n=3 biologically independent experiments). Data were compared using one-way ANOVA with Sidak's multiple comparison test, exact p values are shown.

Figure S2

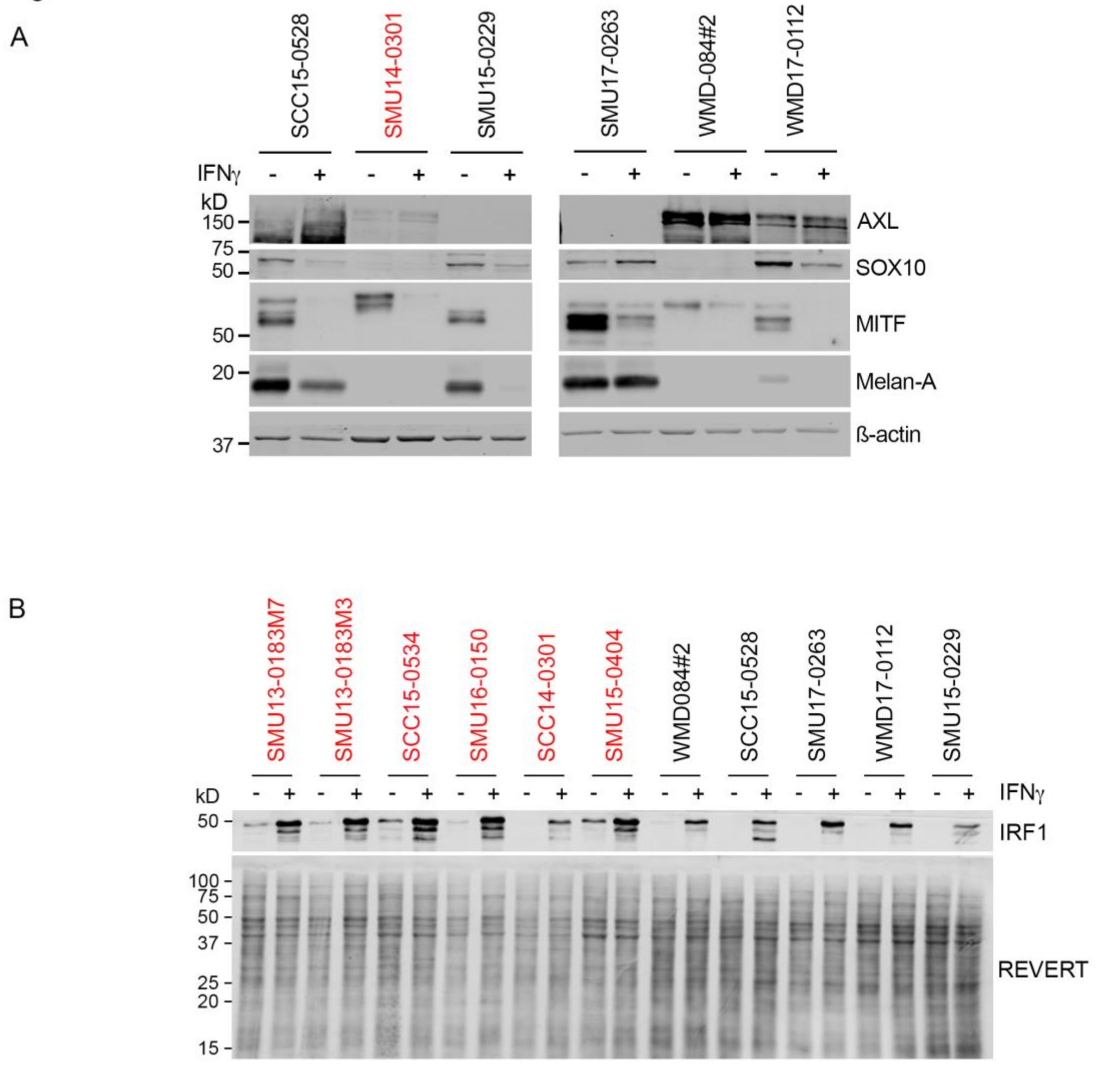

Fig. S2: Persistent IFNγ signaling in PD1 PROG melanoma

A. Accumulation of differentiation markers AXL, SOX10, MITF and Melan-A in a subset of PD1 PROG melanoma cells. SMU14-0301 highlighted in red has intrinsic IFNγ signaling. Remaining PD1 PROG differentiation analysis has been published elsewhere[1]. Experiments repeated independently at least three times.

B. Accumulation of the IRF1 protein in PD1 PROG cell lines (n=11), 24 h after treatment with vehicle control (-) or IFNγ (1000 U/ml). PD1 PROG cell lines with intrinsic IFNγ are indicated in red. Experiments repeated independently at least three times.

Figure S3

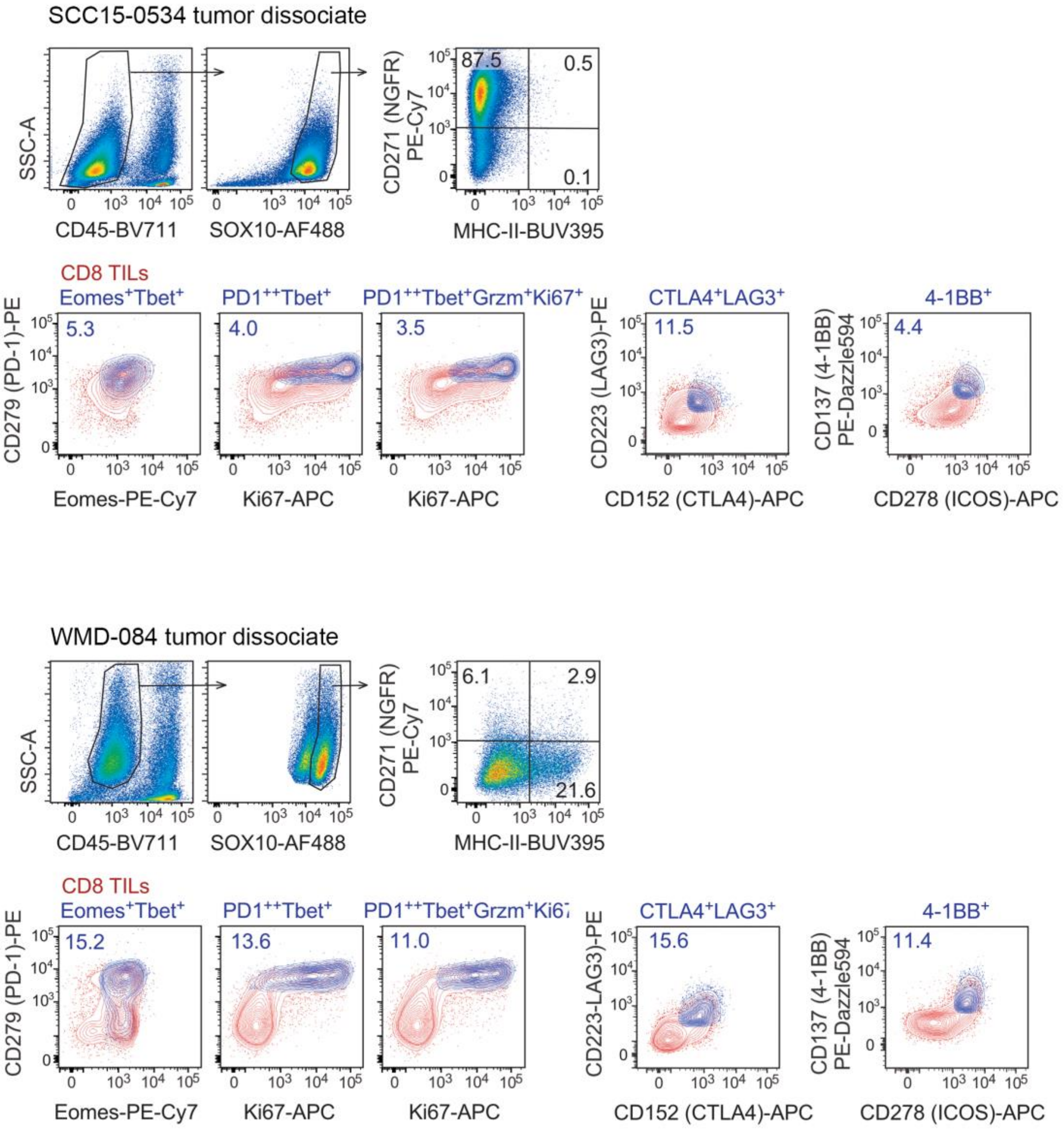

Fig. S3: Persistent IFNγ signaling and immune cell content

Representative flow cytometric profiles of the SMU15-0534 (intrinsic IFNγ signaling) and the WMD-084 (no intrinsic IFNγ signaling) tumor dissociates showing identification of NGFR^{+}/SOX10^{+} melanoma cells (quadrants shows frequency) and antigen experienced PD1^{++} CD8 T cells. CD8 T cells shown in red and subsets of CD8 T cells expressing activation or inhibitory receptors shown in blue, with cell frequencies indicated.

Figure S4

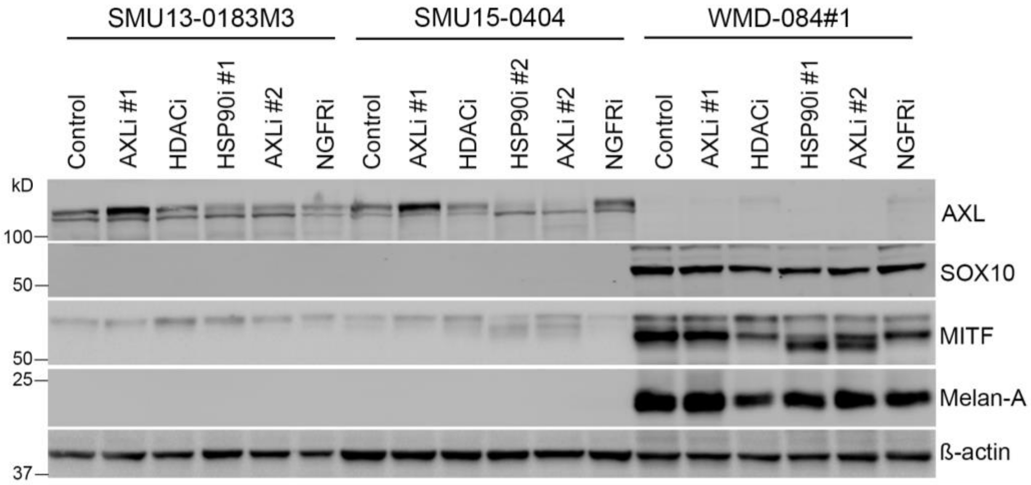

Fig. S4: Restoration of melanoma antigen in de-differentiated PD1 PROG

Accumulation of the AXL, SOX10, MITF and Melan-A protein in the de-differentiated SMU13-0183M3 and SMU15-0404 and the differentiated WMD-084#1, 72h after treatment with AXL inhibitors (AXLi#1, 1 µM R428; AXLi#2 300nM 17-AAG), HDAC inhibitor (HDACi, 25 nM panobinostat), HSP90 inhibitors (HSP90i#1, 250nM Ganetespib or HSP90i#2, 50nM Ganetespib) or NGFR inhibitor (NGFRi, 10µM Tyrphostin AG-879). Experiments repeated independently three times.

Figure S5

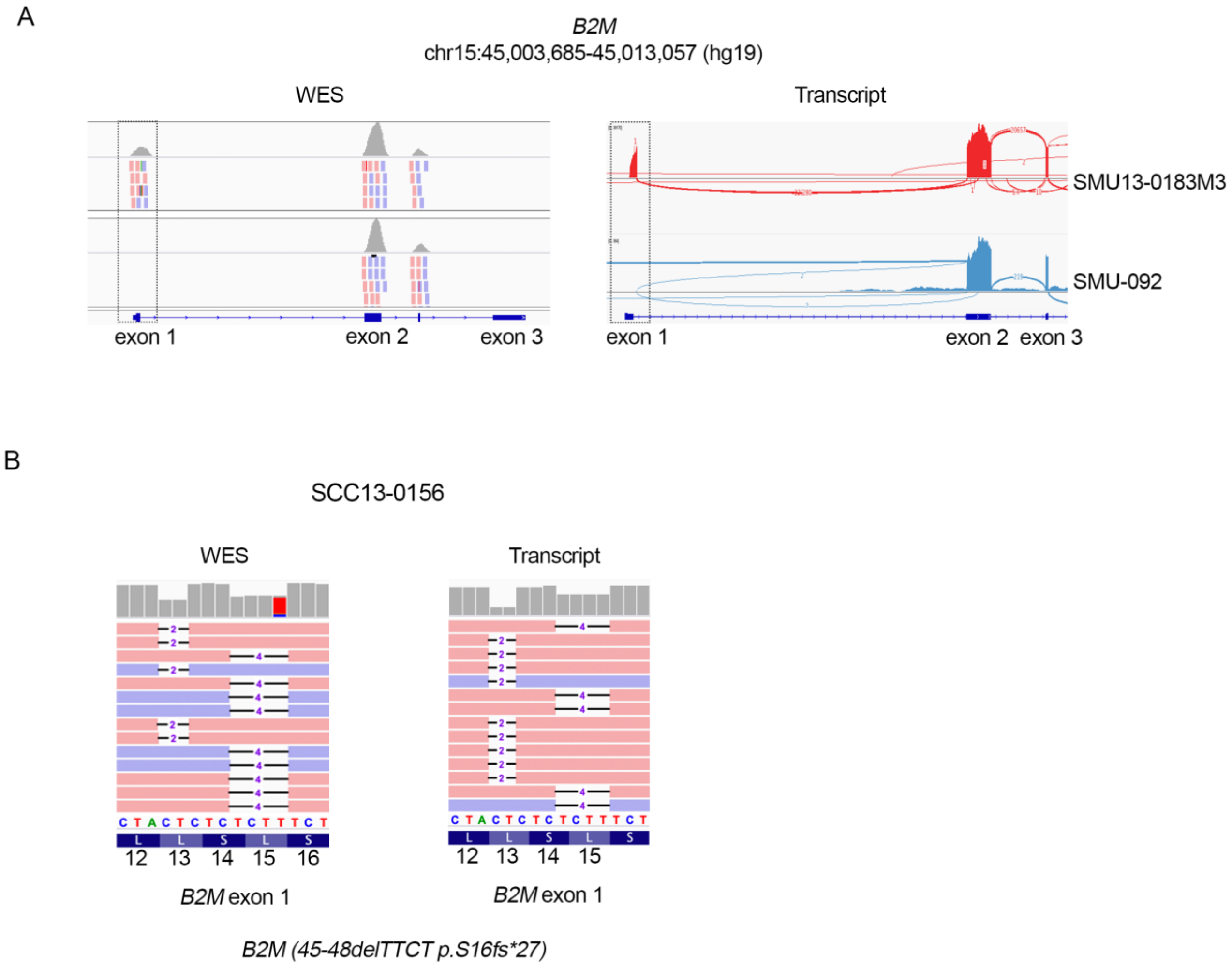

Fig. S5: Whole exome and transcriptome analysis of SMU13-0156 and SMU-092

A. Whole exome (WES) and transcriptome analysis showing a *B2M* exon 1 deletion in SMU-092 compared to SMU13-0183M3.

B. Whole exome and transcriptome analysis of SCC13-0156 showing a frameshift mutation (c45-48delTTCT, p. S16fs*27) in *B2M* exon 1.

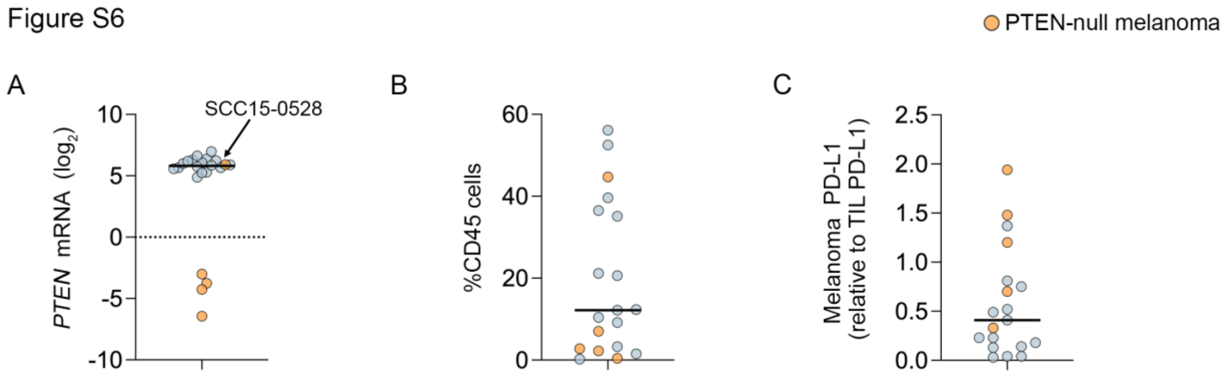

Fig. S6: PTEN loss is associated with immune exclusion

A. *PTEN* transcript expression in PD1 PROG cell lines (n=22). PD1 PROGs highlighted in orange have genetic alterations affecting the *PTEN* locus.

B. Percentage $CD45^{+}$ cells in PD1 PROG tumor dissociates (n=19). Samples corresponding to PD1 PROGs with *PTEN* loss are highlighted in orange.

C. Melanoma PD-L1 (relative to tumor infiltrating lymphocytes (TILs); MFI melanoma/MFI TILs) in PD1 PROG tumor dissociates (n=20). Samples corresponding to PD1 PROGs with *PTEN* loss are highlighted in orange.

Figure S7

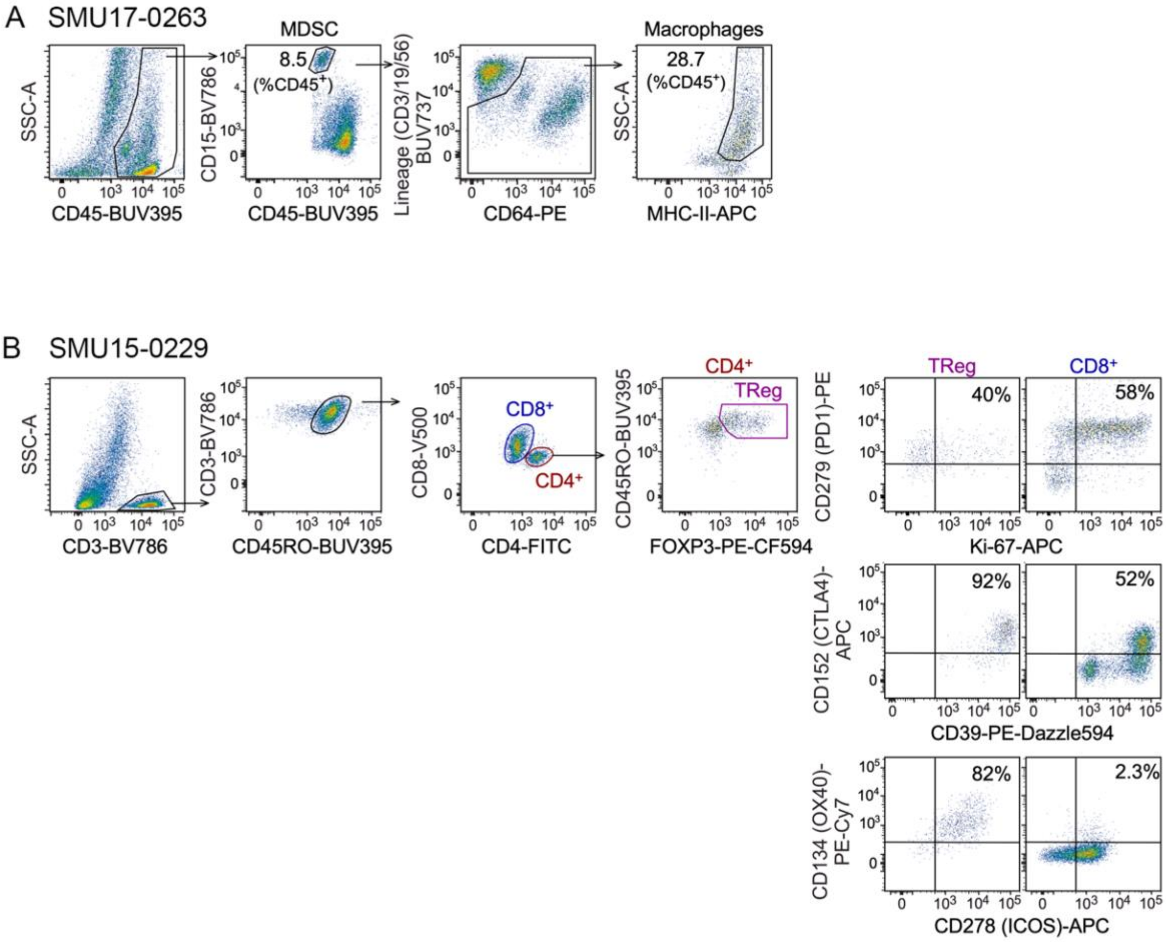

Fig. S7: Tumor microenvironment of MHC-sufficient brain tumors

A. Flow cytometric analysis of the SMU17-0263 tumor dissociate. Frequency of granulocytic myeloid-derived suppressor cells ($CD15^{+}CD45^{int}$ MDSCs) and macrophages ($Lineage^{-}CD64^{+}MHC\text{-}II^{+}$side $scatter^{high}$) is shown as fraction of CD45+ cells.

B. Flow cytometric analysis of the SMU15-0229 tumor dissociate. Phenotype of regulatory T cells (Treg, $CD3^{+}CD45RO^{+}CD4^{+}FOXP3^{+}$) is shown. Phenotype of activated CD8 T cells ($CD3^{+}CD45RO^{+}CD8^{+}$) is shown for comparison.

Figure S8

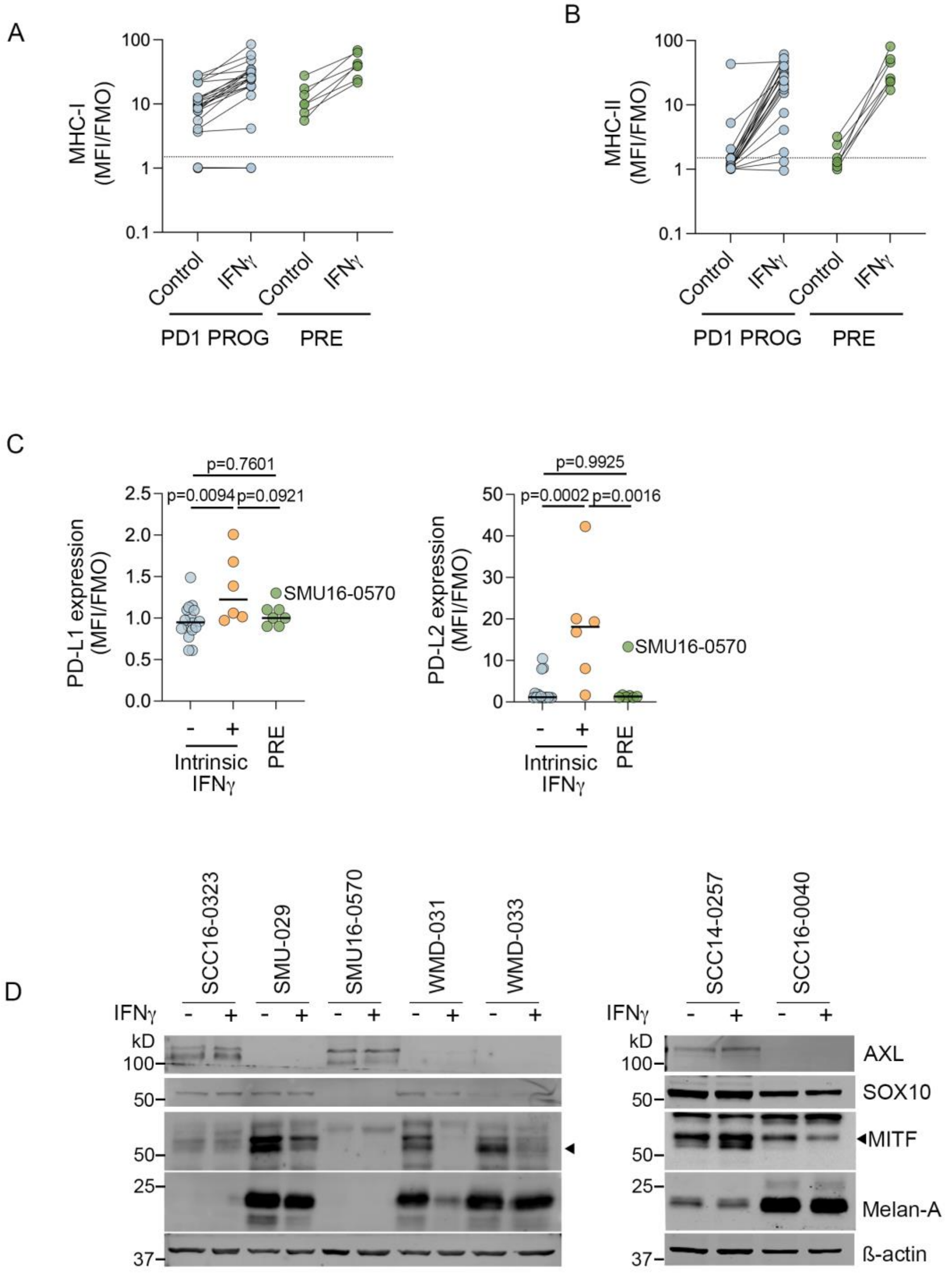

Fig. S8: Immune checkpoint resistance mechanisms are not enriched in pre-treatment melanoma

A, B. Cell surface expression (median fluorescence intensity divided by fluorescence minus one control, MFI/FMO) of MHC-I (A) or MHC-II (B) on PD1 PROG cell lines (n=22) and PRE

melanoma cell lines (n=7), 72 h after treatment with vehicle control or IFNγ (1000 U/ml). Data representative of 3-4 biological replicates.

C. Plots showing relative cell surface expression at baseline (median fluorescence intensity stained divided by fluorescence minus one control, MFI/FMO) of PD-L1 and PD-L2 in PD1 PROGs with intrinsic IFNγ activity (n=6) compared to PD1 PROGs without intrinsic IFNγ activity (n=15) and PRE melanoma cell lines (n=7). Data compared using one-way ANOVA with Tukey's multiple comparison test, adjusted p values are shown.

D. Accumulation of differentiation markers AXL, SOX10, MITF and Melan-A in PRE melanoma cells. Experiments repeated independently at least three times.

Figure S9

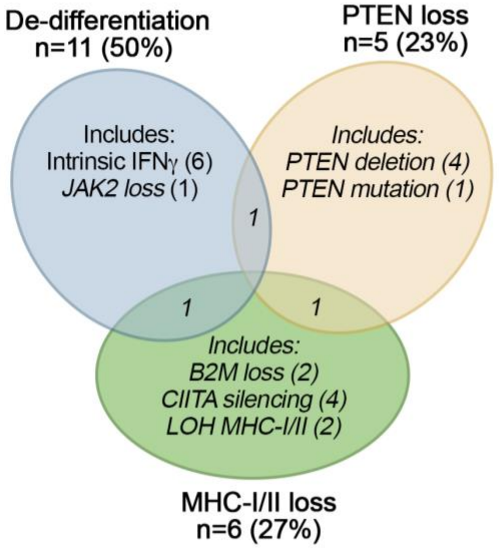

Fig. S9: Mechanisms of melanoma resistance to immune checkpoint inhibitor therapies

Venn diagram showing a summary of the immune checkpoint inhibitor resistance mechanisms identified in the 19/22 PD1 PROG melanoma cell models. LOH, loss of heterozygosity.

Figure S10

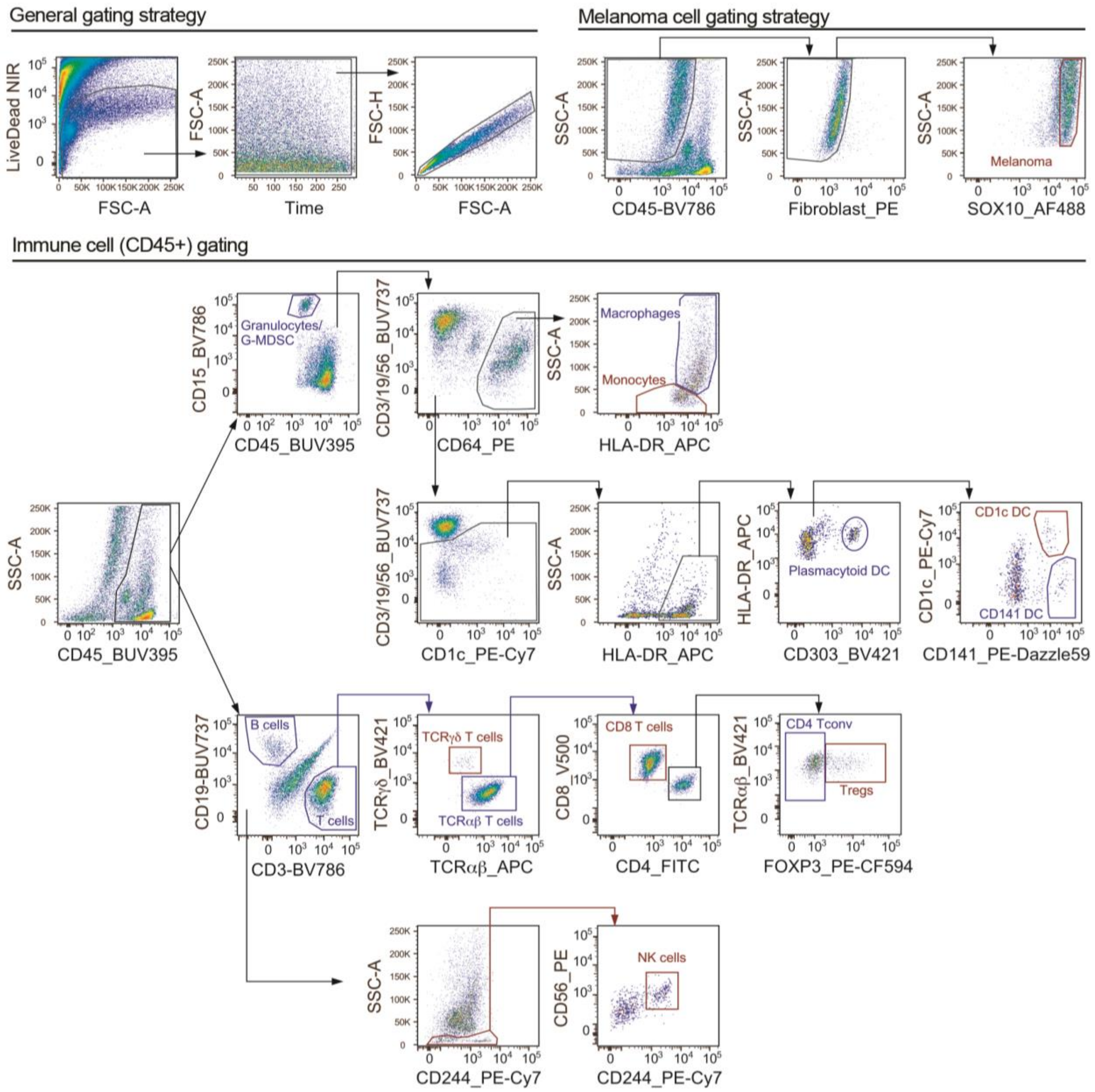

Fig. S10. Flow cytometry gating strategy for tumor dissociate analysis

Top row, general gating strategy (left to right: Live cell gate; Time gate; Single cell gate). Melanoma cells were gated as SSC-A$^{int/high}$CD45^{neg} Fibroblast^{neg}SOX10^{+}.

Immune infiltrating (CD45^{+}) cells were analyzed for myeloid and lymphoid subsets using two separate antibody panels. Myeloid subsets (second and third row) were defined as follows: granulocytes/myeloid-derived suppressor cells (G-MDSCs; CD45^{int}SSC-A^{int}CD15^{+}); tumor-associated macrophages (CD45^{+}CD15^{neg} Lineage(CD3/19/56)^{neg}HLA-DR^{+}CD64^{+}SCC-A^{high}); monocytes (CD45^{+}CD15^{neg}Lineage(CD3/19/56)^{neg}HLA-DR^{+}CD64^{int}SCC-A^{int}); CD45^{+}CD15^{neg}CD64^{neg}Lineage^{neg}cells were further analyzed for plasmacytoid dendritic cells (pDCs, CD45^{+}SCC-A^{low}Lineage^{neg}HLA-DR^{+}CD64^{neg}CD303^{+}); CD141^{+} DCs (CD45^{+}SCC-A^{low}Lineage^{neg}HLA-DR^{+}CD64^{neg}CD303^{neg}CD1c^{neg}CD141^{+}) and CD1c^{+} DCs (CD45^{+}SCC-A^{low}Lineage^{neg}HLA-DR^{+}CD64^{neg} CD303^{neg}CD141^{neg}CD1c^{+}).

Lymphoid subsets (fourth and fifth row) were analyzed for B cells (CD45^{+}SCC-A^{low}CD3^{-}CD19^{+}), T cells (CD45^{+}SCC-A^{low}CD19^{-}CD3^{+}) and natural killer (NK) cells (CD45^{+}SCC-A^{low}CD3^{-}CD19^{-}CD244^{+}CD56^{+}). T cells were further gated for T-cell receptor alpha beta (TCRab; CD45^{+}SCC-A^{low}CD3^{+}TCRgd^{neg}TCRab^{+}) and TCR gamma delta T cells (TCRgd; CD45^{+}SCC-A^{low}CD3^{+}TCRab^{neg}TCRgd^{+}); TCRab T cells were further gated for CD8 T cells (CD45^{+}SCC-A^{low}CD3^{+}TCRab^{+}CD4^{-}CD8^{+}), CD4 T-conventional (Tconv; CD45^{+}SCC-A^{low}CD3^{+} TCRab^{+}CD8^{-}CD4^{+}FOXP3^{-}) and T-regulatory cells (Treg; CD45^{+}SCC-A^{low}CD3^{+}TCRab^{+}CD8^{-}CD4^{+}FOXP3^{+}). Shown is a representative example of tumor dissociate analysis (SMU17-0263).

Supplementary Reference

1 Lee, J. H. *et al.* Transcriptional downregulation of MHC class I and melanoma de-differentiation in resistance to PD-1 inhibition. *Nature Communications* **11**, 1897, doi:10.1038/s41467-020-15726-7 (2020).

Figure S2

A

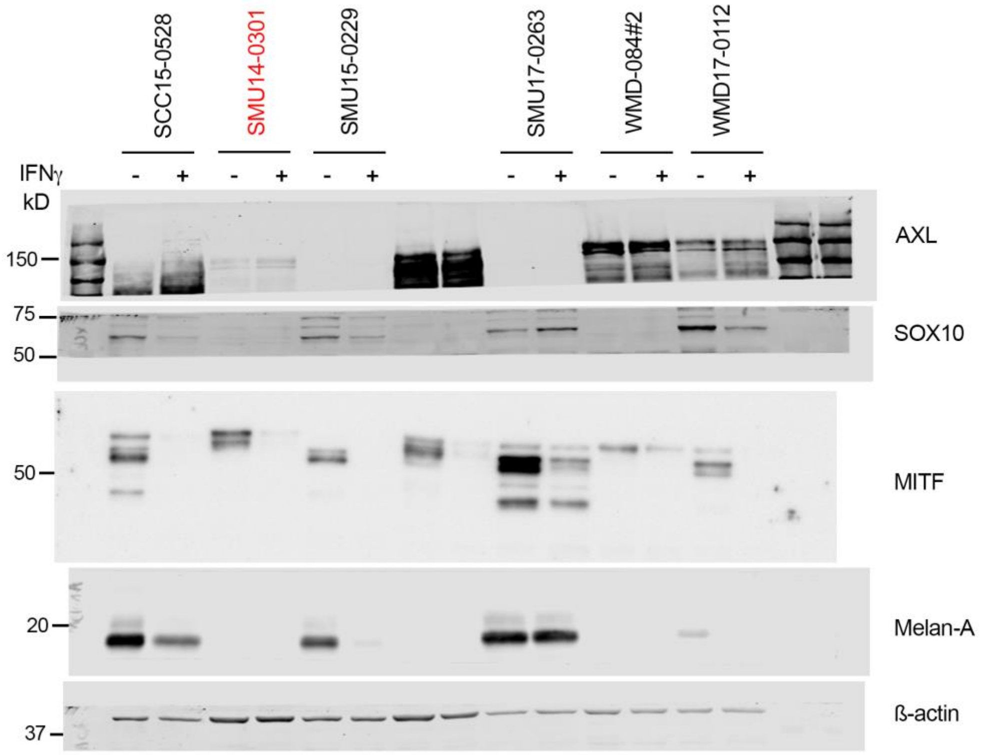

B

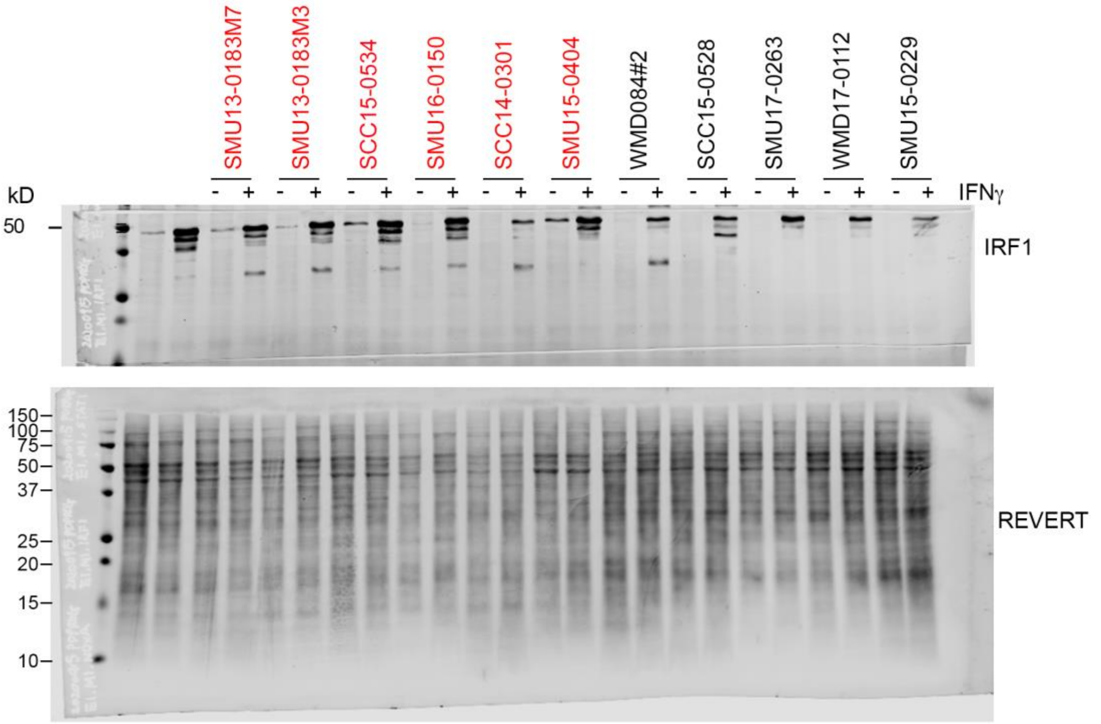

Fig. S2: Uncropped western blots

Figure S4

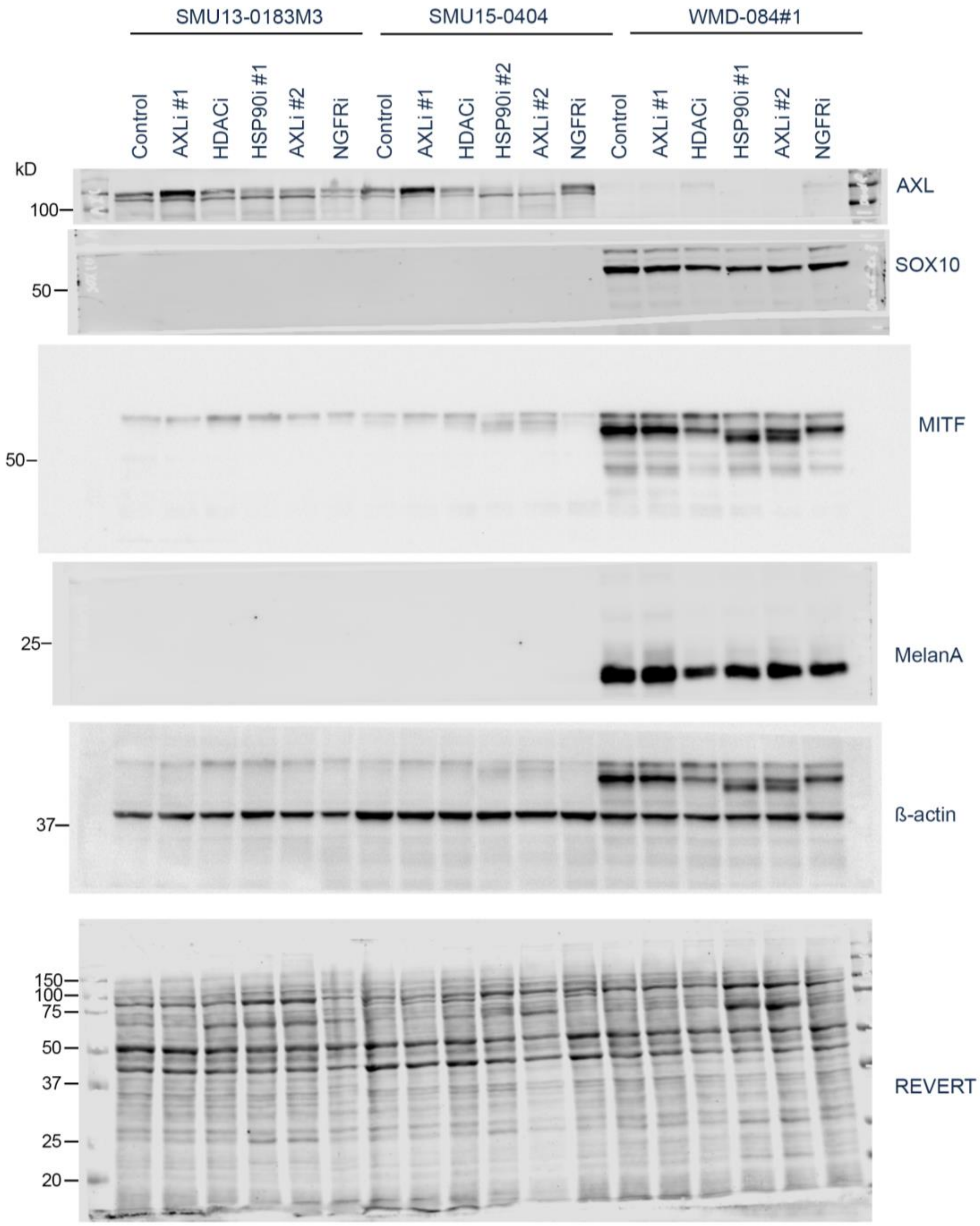

Fig. S4: Uncropped western blots

Figure S8
D

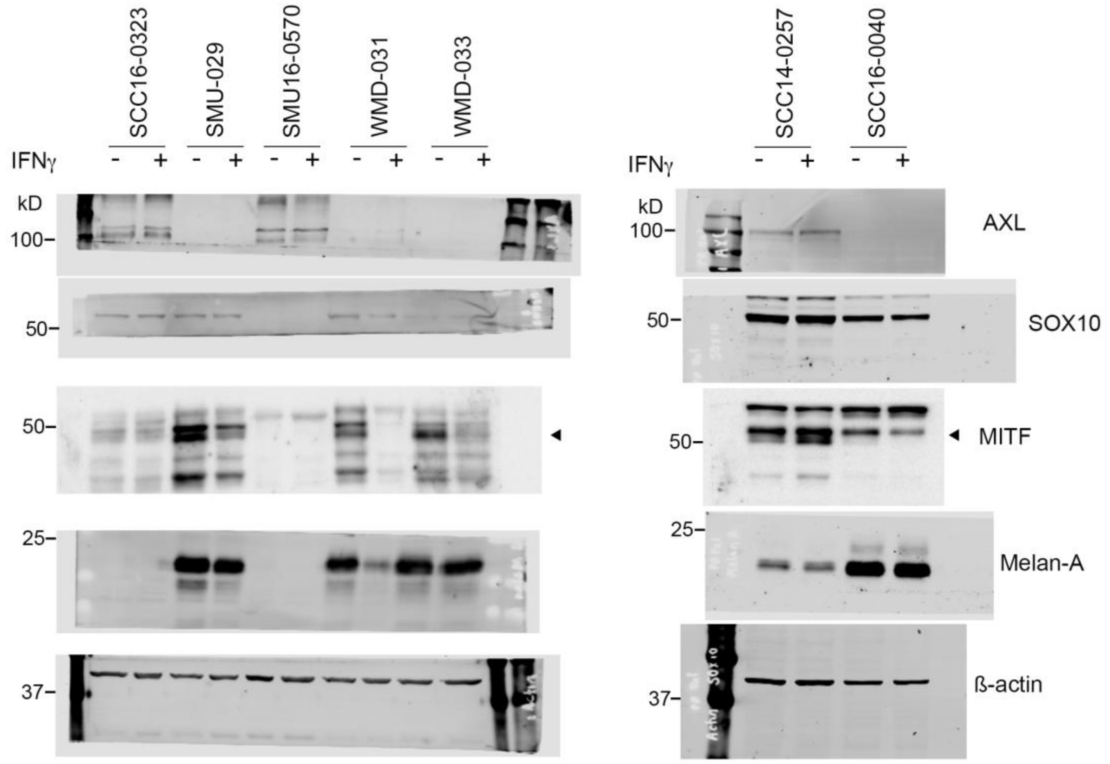

Fig. S8D: Uncropped western blots

Supplementary Table S1: Baseline clinicopathologic characteristics of melanoma patients

Characteristics	Patients (n=18)
Age, median (range)[a]	65.5 (31-81)
Sex, n (%)	
Male	6 (33)
Female	12 (67)
Treatment	
Single agent PD1	9 (50)
Combination PD1 and CTLA4	9 (50)
Prior therapy	
Yes	10 (56)
No	8 (44)
M Stage (AJCC 8th edition), n (%)	
M1b	3 (17)
M1c	7 (39)
M1d	8 (44)
Mutation, n (%)	
$BRAF^{V600}$	10 (56)
NRAS	6 (33)
Other[b]	2 (11)
LDH at baseline, n (%)	
≤ ULN	11 (61)
> ULN	6 (33)
Unknown	1 (6)
Response (RECIST 1.1), n (%)	
PR	6 (33)
SD	3 (17)
PD	9 (50)

[a]Age at time of PD1 PROG biopsy resection

[b]One patient had a BRAF G469E mutation and another had an NF1 truncating mutation.

Abbreviations: ULN, upper limit of normal; PR, partial response; SD, stable disease; PD, progressive disease

Supplementary Table S2: Detailed characteristics of patients and PD1 PROG melanoma cell lines used in this study

Patient ID	PD1 PROG	Sex	Disease stage at treatment	Mutation status	Prior therapy	LDH at baseline	Treatment	Clinical trial	irRC	Type of disease progression	Site of biopsy	Treatment at time of biopsy	On treatment at time of biopsy
SCC11-0270	SCC11-0270	M	M1d	BRAF V600E	BRAFi + MEKi (dabrafenib + trametinib)	Normal	Nivolumab 3mg/kg q2w	ABC BMS CA209-170	PD	Innate	Brain	Nivolumab	Yes
SCC13-0156	SCC13-0156	F	M1c	BRAF V600E	1. anti-PD1 (pembrolizumab) 2. BRAFi + MEKi + LEEi (binimetinib, ribociclib, encorafenib)	Normal	Nivolumab 3mg/kg q2w		SD	Acquired	Retroperitoneal LN	Nivolumab	Yes
SCC15-0111	SCC15-0111	M	M1c	BRAF V600K	BRAFi + MEKi (dabrafenib + trametinib)	Normal	Pembrolizumab 2mg/kg q3w		PR	Innate	Brain	Pembrolizumab	Yes
SCC15-0528	SCC15-0528	M	M1b	BRAF V600E	BRAFi + MEKi (dabrafenib + trametinib)	Normal	Ipilimumab 3mg/kg+ nivolumab 1mg/kg q3w	BMS CA209-511	PD	Innate	Thigh subcutaneous	Ipilimumab + nivolumab	Yes
SCC15-0534	SCC15-0534	F	M1b	NRAS Q61K	None	Normal	Pembrolizumab 2mg/kg		PR	Acquired	Neck	Pembrolizumab	Yes
SMU-059	SMU-059	F	M1c	NRAS Q61R	Ipilimumab	> 1x ULN	Pembrolizumab 2mg/kg q3w		PD	Innate	Flank subcutaneous	Pembrolizumab	Yes
SMU-092	SMU-092	F	M1d	NRAS Q61L	None	> 1x ULN	Ipilimumab 3mg/kg + nivolumab 1mg/kg q3w	ABC BMS CA209-170	PR	Acquired	Breast	None	No
SMU11-0376	SMU11-0376M2 SMU11-0376M4	F	M1d	BRAF V600E	BRAFi + MEKi (dabrafenib + trametinib)	Normal	Nivolumab 3mg/kg q2w	ABC BMS CA209-170	PD	Innate	Brain	Nivolumab	Yes
SMU13-0183	SMU13-0183M3 SMU13-0183M7	F	M1d	BRAF V600E	BRAFi + MEKi (dabrafenib + trametinib)	Normal	Nivolumab 3mg/kg q2w	ABC BMS CA209-170	PD	Innate	Brain	Nivolumab	Yes
SMU14-0301	SMU14-0301	F	M1c	BRAF V600E	BRAFi + MEKi (dabrafenib + trametinib)	> 1x ULN	Pembrolizumab 10mg/kg q2w	MK3475-006	SD	Acquired	Retroperitoneal LN	Pembrolizumab	Yes
SMU15-0229	SMU15-0229	M	M1c	BRAF V600E	BRAFi + MEKi (dabrafenib + trametinib)	> 2x ULN	Ipilimumab 3mg/kg+ nivolumab 1mg/kg q3w		PD	Innate	Brain	None	No
SMU15-0404	SMU15-0404	F	M1d	BRAF G469R	None	Normal	Pembrolizumab 120 mg q3w		SD	Innate	Axillary LN	Pembrolizumab	Yes

SMU16-0150	SMU16-0150	F	M1d	BRAF V600K	None	Normal	Ipilimumab 3mg/kg + nivolumab 1mg/kg	ABC BMS CA209-170	PD	Innate	Scalp	None	No
SMU17-0263	SMU17-0263	M	M1d	NF1 R1241*	None	Unknown	Ipilimumab 3mg/kg + nivolumab 1mg/kg q3w		PD	Innate	Brain	None	No
WMD-084	WMD-084#1<br>WMD-084#2	F	M1c	NRAS Q61K	None	> 2x ULN	Ipilimumab 1mg/kg + Pembrolizumab 2mg/kg q3w	MK3475-029	PR	Acquired	Ovaries	Pembrolizumab	Yes
WMD15-083	WMD15-083#1<br>WMD15-083#2	F	M1c	NRAS Q61K	None	Normal	Ipilimumab 1mg/kg + pembrolizumab 2mg/kg q3w	MK3475-029	PR	Acquired	Small bowel<br>Large colon	None	No
WMD17-0112	WMD17-0112	F	M1d	BRAF V600E	1. anti-PD1 + anti-IDO1 (nivolumab + BMS-986205)<br>2. BRAFi + MEKi (dabrafenib + trametinib)	> 1x ULN	Ipilimumab 3mg/kg + nivolumab 1mg/kg q3w		PD	Innate	Thigh subcutaneous	Nivolumab	Yes
SCC16-0016	SCC16-0016	M	M1b	NRAS Q61K	None	Normal	Ipilimumab 1mg/kg + pembrolizumab 2mg/kg q3w	MK3475-029	PR	Acquired	Pancreas	Pembrolizumab	Yes

irRC: immune-related RECIST criteria; CR, complete response; PR, partial response; SD, stable disease; PD, progressive disease; Sex: M, male; F, female; Biopsy site: LN, lymph node; SC, subcutaneous; LDH, lactate dehydrogenase, PROG, progressing, ULN, upper limit of normal

Supplementary Table S3: Detailed characteristics of PRE-treatment melanoma cell lines used in this study

Patient ID	PRE-treatment cell line	Sex	Disease stage at treatment	Mutation status	Prior therapy	LDH at baseline	Subsequent immunotherapy	irRC	Site of biopsy
SCC14-0257	SCC14-0257	F	IIIC	BRAF V600K	None	Normal	None	NA	Shin
SCC16-0040	SCC16-0040	M	IIIB	BRAF V600K	None	Normal	Ipilimumab 3mg/kg + nivolumab 1mg/kg q3w	PR	Parotid LN
SCC16-0323	SCC16-0323	F	III	BRAF L597S, PTEN D92Y*	None	Normal	None	NA	Axilla LN
SMU-029	SMU-029	M	III (intransit)	BRAF V600E	None	Normal	None	NA	Calf
SMU16-0570	SMU16-0570	M	M1d	PTEN p.A192fs*20	None	> 1x ULN	Ipilimumab 1mg/kg + nivolumab 3mg/kg q3w	PR	Frontal lobe
WMD-031	WMD-031	F	M1a	BRAF V600E, PTEN A126S**	None	> 1x ULN	None	NA	Axilla LN
WMD-033	WMD-033	M	M1c	BRAF V600E	None	> 1x ULN	Pembrolizumab 2mg/kg (1 dose only)	NA	Thigh

irRC: immune-related RECIST criteria; PR, partial response; Sex: M, male; F, female; Biopsy site: LN, lymph node; LDH, lactate dehydrogenase; ULN, upper limit of normal; NA, not available

*PTEN D92Y dbSNP1554898067 has unknown clinical significance according to ClinVar

**PTEN A126S dbSNP1554898129 has unknown clinical significance according to ClinVar

Supplementary Table S4: List of flow cytometry antibodies

Antibody	Cat No	Company	Dilution	Comment
Beta-2-microglobulin PE-Cy7	316318	Biolegend	1/200	
CD1c (BDCA-1) PE-Cy7	331516	Biolegend	1/40	
CD3 AlexaFluor700	300424	Biolegend	1/100	
CD3 BV786	565491	BD Horizon	1/100	
CD3 BUV737	564308	BD Horizon	1/100	
CD4 FITC	344604	Biolegend	1/100	1/200 in vitro
CD4 AlexaFluor700	357418	Biolegend	1/40	
CD8 V500	561617	BD Horizon	1/100	1/200 in vitro
CD15 BV786	741013	BD OptiBuild	1/200	
CD16 AlexaFluor700	557920	BD Pharmingen	1/50	
CD19 BUV737	564303	BD Horizon	1/50	
CD38 PE-Cy7	356608	Biolegend	1/100	
CD39 PE-Dazzle594	328224	Biolegend	1/100	
CD45 BV786	563716	BD Horizon	1/50	
CD45 BUV395	563792	BD Horizon	1/80	
CD45RA BUV737	564442	BD Horizon	1/100	
CD45RO BUV395	564291	BD Horizon	1/30	
CD56 PE	130-113-312	Miltenyi Biotech	1/50	
CD64 PE	305007	Biolegend	1/100	
CD69 APC	555533	BD Pharmingen	1/20	
CD103 PE	350206	Biolegend	1/40	
CD107a PE	555801	BD Pharmingen	1/10	Intracellular
CD134 (OX-40) PE-Cy7	563663	BD Pharmingen	1/20	
CD137 (4-1BB) PE-Dazzle594	309826	Biolegend	1/20	
CD141 PE-Dazzle594	344120	Biolegend	1/40	
CD152 (CTLA-4) APC	17-1529-42	Thermo Fisher Scientific (eBioscience)	1/20	
CD223 (LAG3) PE	130-105-452	Miltenyi Biotech	1/11	
CD244 PE-Cy7	329520	Biolegend	1/80	
CD271 (NGFR) PE-Cy7	345110	Biolegend	1/100	
CD273 (PD-L2) APC	329608	Biolegend	1/20	1/50 cell lines
CD274 (PD-L1) BV421	329714	Biolegend	1/50	1/40 cell lines
CD278 (ICOS) APC	313510	Biolegend	1/40	
CD279 (PD-1) BV421	562516	BD Horizon	1/40	
CD303 BV421	566427	BD Horizon	1/40	
EOMES PE-Cy7	25-4877-42	Thermo Fisher Scientific (eBioscience)	1/20	
Fc block	564220	BD	1/200	
FLAG APC	130-119-683	Miltenyi Biotech	1/50	
Fibroblast PE	130-100-136	Miltenyi Biotech	1/11	
Fixable Near-IR Dead Cell Stain	L34976	Thermo Fisher Scientific	1/100	
FOXP3 AlexaFluor488	320212	Biolegend	1/20	Intracellular
FOXP3 PE-CF594	563955	BD Horizon	1/20	Intracellular
Granzyme B AlexaFluor700	560213	BD Pharmingen	1/100	Intracellular
HLA-A2 PE-Cy7	561347	BD Pharmingen	1/100	
HLA-A, B, C AlexaFluor 700	311438	Biolegend	1/80	
HLA-A, B, C PE	311406	Biolegend	1/100	
HLA-DR FITC	307604	Biolegend	1/100	
HLA-DR APC	307610	Biolegend	1/80	
HLA-DR, DP, DQ BUV395	740302	BD OptiBuild	1/150	
Human IgG4-Fc PE	9200-09	Southern Biotech	1/100	Pembrolizumab detection

IFNgamma AF647	563495	BD Pharmingen	1/20	Intracellular
Isotype control	400202	Biolegend	as indicated	In vitro blocking
KI67 APC	17-5699-42	Thermo Fisher Scientific (eBioscience)	1/200	Intracellular
Pembrolizumab		Merck	20ug/ml	
SOX10 AlexaFluor488	sc-365692 AF488	Santa Cruz	1/50	Intracellular
TBET BV421	563318	BD Horizon	1/20	Intracellular
TCRalphabeta APC	306718	Biolegend	1/100	
TCRgammadelta BV421	744870	BD Optibuild	1/80	

Supplementary Table S5: Flow cytometry gating strategy for cell subset identification

Subset	Identification[1]	Further characterization
Melanoma	CD45-neg_SCC-A-int to high_Fibroblast-neg_SOX-10+	MHC class I (HLA-A, B, C)_MHC class II (HLA-DR, DP, DQ)_CD146/MCAM_CD271/NGFR_CD274/PD-L1_CD273/PD-L2
Tumor-infiltrating lymphocytes (TILs)	CD45+_SCC-A-low_CD3+	MHC class I (HLA-A, B, C)_MHC class II (HLA-DR, DP, DQ)_CD274/PD-L1_CD273/PD-L2
Granulocytes/myeloid-derived suppressor cells	CD45-int_SCC-A-int_CD15+	
Tumor-associated macrophages (TAMs)	CD45+_SCC-A-high_CD3-neg-CD19-neg_HLA-DR+_CD64+	
Monocytes	CD45+_SCC-A-int_CD3-neg-CD19-neg_HLA-DR+_CD64-int	
Plasmacytoid dendritic cells (pDC)	CD45+_SCC-A-low_CD3-neg-CD19-neg_HLA-DR+_CD64-neg_CD303+	
CD141+ dendritic cells	CD45+_SCC-A-low_CD3-neg-CD19-neg_HLA-DR+_CD64-neg_CD303-neg_CD1c-neg_CD141+	
CD1c+ dendritic cells (mo-DC)	CD45+_SCC-A-low_CD3-neg-CD19-neg_HLA-DR+_CD64-int_CD303-neg_CD141-neg_CD1c+	
T cells, TCR alpha beta	CD45+_SCC-A-low_CD3+_TCRab+	
T cells, TCR gamma delta	CD45+_SCC-A-low_CD3+_TCRgd+	
CD8 T cells	CD45+_SCC-A-low_CD3+_TCRab+_CD4-neg_CD8+; alternative gating CD45+ or CD45RO+_SCC-A-low_CD3+_CD4-neg_CD8+	[CD279/PD-1_Proliferation (Ki-67)_EOMES_TBET_Granzyme B] or [CD278_CD137_CD134]
Conventional CD4 T cells (Tconv)	CD45+_SCC-A-low_CD3+_TCRab+_CD8-neg_CD4+_FOXP3-neg; alternative gating CD45+ or CD45RO+_SCC-A-low_CD3+_CD8-neg_CD4+_FOXP3-neg	[CD279/PD-1_Proliferation (Ki-67)_EOMES_TBET_Granzyme B] or [CD278_CD137_CD134]
Regulatory T cells (Tregs)	CD45+_SCC-A-low_CD3+_TCRab+_CD8-neg_CD4+_FOXP3+; alternative gating CD45+ or CD45RO+_SCC-A-low_CD3+_CD8-neg_CD4+_FOXP3+	[CD279/PD-1_Proliferation (Ki-67)_EOMES_TBET_Granzyme B] or [CD278_CD137_CD134]
B cells	CD45+_SCC-A-low_CD3-neg_CD19+	
NK cells	CD45+_SCC-A-low_CD3-neg_CD244+_CD56+/int	CD16

[1]General gating (applies to all): Non-Debris (exclude FCS-A-low/SSC-A-low_Live cells (LiveDead-neg)_Time gate_Single cells (FCS-A/FCS-H)
