# Supplementary material for: The molecular and functional landscape of resistance to immune checkpoint blockade in melanoma
Source: Nat Commun. 2023 Mar 18;14:1516. doi: 10.1038/s41467-023-36979-y (PMC10024679; doi:10.1038/s41467-023-36979-y)
Supplement: Supplementary file 3 — Reporting Summary [file 41467_2023_36979_MOESM3_ESM.pdf]

nature portfolio

Corresponding author(s): Rizos

Last updated by author(s): 26/10/22

Reporting Summary

Nature Portfolio wishes to improve the reproducibility of the work that we publish. This form provides structure for consistency and transparency in reporting. For further information on Nature Portfolio policies, see our Editorial Policies and the Editorial Policy Checklist.

Statistics

For all statistical analyses, confirm that the following items are present in the figure legend, table legend, main text, or Methods section.

n/a	Confirmed	
☐	☒	The exact sample size (*n*) for each experimental group/condition, given as a discrete number and unit of measurement
☐	☒	A statement on whether measurements were taken from distinct samples or whether the same sample was measured repeatedly
☐	☒	The statistical test(s) used AND whether they are one- or two-sided<br>*Only common tests should be described solely by name; describe more complex techniques in the Methods section.*
☐	☒	A description of all covariates tested
☐	☒	A description of any assumptions or corrections, such as tests of normality and adjustment for multiple comparisons
☐	☒	A full description of the statistical parameters including central tendency (e.g. means) or other basic estimates (e.g. regression coefficient) AND variation (e.g. standard deviation) or associated estimates of uncertainty (e.g. confidence intervals)
☐	☒	For null hypothesis testing, the test statistic (e.g. *F*, *t*, *r*) with confidence intervals, effect sizes, degrees of freedom and *P* value noted<br>*Give P values as exact values whenever suitable.*
☒	☐	For Bayesian analysis, information on the choice of priors and Markov chain Monte Carlo settings
☒	☐	For hierarchical and complex designs, identification of the appropriate level for tests and full reporting of outcomes
☒	☐	Estimates of effect sizes (e.g. Cohen's *d*, Pearson's *r*), indicating how they were calculated

Our web collection on statistics for biologists contains articles on many of the points above.

Software and code

Policy information about availability of computer code

Data collection	Sequencing data was generated with Illumina sequencing instruments. No specialized software was developed for data collection.
Data analysis	RNA sequencing data were processed as described in Lee et al. Nature Communications 11, 1897, doi:10.1038/s41467-020-15726-7 (2020), using cutadapt (v1.9), STAR version 2.5.2, GenomicAligments version 1.12.2<br>Bioconductor version 3.9 (BiocManager 1.30.4), R 3.6.0 (2019-04-26)<br>Limma version 3.52.4<br>RSEM was used to derive the FPKM estimates using GENCODE Genes version 26<br>Absolute single sample gene set enrichment analysis (GSEA) implementation by GenePattern<br>GSEA pre-ranked (version 7.4.0) provided by GenePattern (https://cloud.genepattern.org/)<br>FPKM values were also used to infer the relative proportions of 22 types of infiltrating immune cells using the CIBERSORT web portal (http://cibersort.stanford.edu/).<br>Nearest neighbor algorithm within the Morpheus web based tool (https://software.broadinstitute.org/morpheus/).<br>QIAGEN Digital Insights (Software Build: 9.0.0.20220826; https://variants.ingenuity.com/qci/) was used for whole exome sequence variant analysis<br>Statistical analysis using GraphPad Prism version 9

For manuscripts utilizing custom algorithms or software that are central to the research but not yet described in published literature, software must be made available to editors and reviewers. We strongly encourage code deposition in a community repository (e.g. GitHub). See the Nature Portfolio guidelines for submitting code & software for further information.

Data

Policy information about availability of data

All manuscripts must include a data availability statement. This statement should provide the following information, where applicable:

- Accession codes, unique identifiers, or web links for publicly available datasets
- A description of any restrictions on data availability
- For clinical datasets or third party data, please ensure that the statement adheres to our policy

The RNAseq data is deposited in Sequence Read Archive (BioProject PRJNA818797) and can be accessed by reviewers using the following link: https://dataview.ncbi.nlm.nih.gov/object/PRJNA818797?reviewer=jhjus1vbtf7qq8unt4ure3ug75
RNA sequencing data were mapped to reference genome hg19 (GRCh37, https://www.ncbi.nlm.nih.gov/assembly/GCF_000001405.13/) or hg38 (GRCh38, https://www.ncbi.nlm.nih.gov/assembly/GCF_000001405.26/)
All other data is available within the Article, Supplementary Information or Source data files.

Human research participants

Policy information about studies involving human research participants and Sex and Gender in Research.

Reporting on sex and gender	Patient sex was included in this study, based on self-reporting, and reported in Table S1 and S2. Sex-based analyses was not performed as this was not part of the objectives of this study. Written consent was obtained from all patients (Human Research ethics committee protocols from Royal Prince Alfred Hospital; Protocol X15-0454 & HREC/11/RPAH/444).
Population characteristics	Patient characteristics in this study include age, sex, treatment type, prior treatment status, disease stage, LDH levels, mutation status, RECIST response, and type of disease progression. All patients received PD-1 inhibitors ± CTLA4 inhibitor; 56% had prior treatment. Median age of population is 65.5 (range 31-81), 33% male, 56% BRAFV600-mutant and 33% NRAS-mutant.
Recruitment	This study included metastatic melanoma patients who were treated and progressed on treatment with immune checkpoint inhibitors (ie PD-1 inhibitors (pembrolizumab or nivolumab) ± CTLA4 inhibitor, ipilimumab at Melanoma Institute Australia (MIA) and affiliated hospitals. Patients with progressive disease while on immune checkpoint inhibitor treatment and had resected fresh tumor tissues at the time of immune checkpoint inhibitor progression, of which short-term melanoma cell models were generated, were included in this study; there were no self selection bias.
Ethics oversight	Written consent was obtained from all patients (Human Research ethics committee protocols from Royal Prince Alfred Hospital; Protocol X15-0454 & HREC/11/RPAH/444).

Note that full information on the approval of the study protocol must also be provided in the manuscript.

Field-specific reporting

Please select the one below that is the best fit for your research. If you are not sure, read the appropriate sections before making your selection.

[x] Life sciences [ ] Behavioural & social sciences [ ] Ecological, evolutionary & environmental sciences

For a reference copy of the document with all sections, see nature.com/documents/nr-reporting-summary-flat.pdf

Life sciences study design

All studies must disclose on these points even when the disclosure is negative.

Sample size	While no formal power calculations were used to determine sample sizes, across all expreriments, sample sizes were selected to ensure robust statistical analysis within the confines and technical parameters of each experiment. Cell experiments were completed with n=3+ biological replicates, and human tissue samples that passed quality control were included for sequencing and flow cytometry analyses.
Data exclusions	No data excluded from analyses
Replication	Cell experiments were performed using at least three biological experiments unless otherwise stated. The individual data points are displayed in each figure. When technical replicates were included within experiments, these are detailed in the relevant figures.<br>Flow cytometry analysis of patient biopsies were only performed once due to the limited samples. Next generation sequencing replications were not performed due to study costs and limited material. Transcriptome analyses were performed comparing groups (e.g melanoma tumors with (n=6) vs without (n=15) intrinsic IFNg activity) to mitigate risks of sequencing errors. DNA sequencing findings were validated with capillary sequencing.
Randomization	Samples were allocated into experimental groups based on transcriptome signatures or treatment (i.e. control vs IFNg treated; melanoma tumors with vs without intrinsic IFNg activity). RNA sequencing and flow cytometry experiments were performed based upon sample and data availability. The cohort of patients were already randomized as they were participants in clinical trials.

Blinding

The investigators were blinded to the response annotations until they became available. Flow cytometry scoring was performed prior to the analysis of the results. Otherwise blinding was not utilized in this study as it was not feasible for the mammalian cell culture and transcriptome experiments.

Reporting for specific materials, systems and methods

We require information from authors about some types of materials, experimental systems and methods used in many studies. Here, indicate whether each material, system or method listed is relevant to your study. If you are not sure if a list item applies to your research, read the appropriate section before selecting a response.

Materials & experimental systems

n/a	Involved in the study
☐	☒ Antibodies
☐	☒ Eukaryotic cell lines
☒	☐ Palaeontology and archaeology
☒	☐ Animals and other organisms
☐	☒ Clinical data
☒	☐ Dual use research of concern

Methods

n/a	Involved in the study
☒	☐ ChIP-seq
☐	☒ Flow cytometry
☒	☐ MRI-based neuroimaging

Antibodies

Antibodies used

All antibodies used in this study includig supplier name, catalog number and clone number are provided in Table S4 or detailed in the Methods section and summarised here.
Antibodies for Flow cytometry

Antibody,Cat No,Company,Dilution, Clone, Lot number
Beta-2-microglobulin PE-Cy7,316318,Biolegend,1/200, Clone 2M2, Lot B250481
CD1c (BDCA-1) PE-Cy7,331516,Biolegend,1/40, Clone L161, Lot B250481
CD3 AlexaFluor700,300424, Biolegend, 1/100, Clone UCHT1, Lot B244777
CD3 BV786,565491,BD Horizon,1/100, Clone UCHT1, Lot 9336141
CD3 BUV737,564308,BD Horizon,1/100, Clone UCHT1, Lot 7005838; 7335654
CD4 FITC,344604,Biolegend,1/100, Clone SK3, Lot B244280
CD4 AlexaFluor700,357418, Biolegend,1/40, Clone A161A1, Lot B247090
CD8 V500,561617,BD Horizon,1/100, Clone SK1, Lot 8288536; 8003887; 9268333
CD15 BV786,741013,BD OptiBuild,1/200, Clone W6D3, Lot 7248954
CD16 AlexaFluor700,557920,BD Pharmingen,1/50, Clone 3G8, Lot 7082769
CD19 BUV737,564303,BD Horizon,1/50, Clone SJ25C1, Lot 8344734
CD38 PE-Cy7,356608,Biolegend,1/100, Clone HB-7, Lot B247239
CD39 PE-Dazzle594,328224,Biolegend,1/100, Clone A1, Lot B250792
CD45 BV786,563716,BD Horizon,1/50, Clone HI30, Lot 8012943
CD45 BUV395,563792,BD Horizon,1/80, Clone HI30, Lot 7103721; 9269807
CD45RA BUV737,564442,BD Horizon,1/100, Clone HI100, Lot 7075872
CD45RO BUV395,564291,BD Horizon,1/30, Clone UCHL1, Lot 7282866; 8094773
CD56 PE,130-113-312,Miltenyi Biotech,1/50, Clone REA196, Lot 5190320350; 5200603191
CD64 PE,305007,Biolegend,1/100, Clone 10.1, Lot B241143
CD69 APC,555533,BD Pharmingen,1/20, Clone FN50, Lot 8150729
CD103 PE,350206,Biolegend,1/40, Clone Ber-ACT8, Lot B242620
CD107a PE,555801,BD Pharmingen,1/10, Clone H4A3, Lot 8025951; 8130821
CD134 (OX-40) PE-Cy7,563663,BD Pharmingen,1/20, Clone Ber-ACT35, Lot 7104547
CD137 (4-1BB) PE-Dazzle594,309826,Biolegend,1/20, Clone 4B4-1, Lot B253152
CD141 PE-Dazzle594,344120,Biolegend,1/40, Clone M80, Lot B236210
CD152 (CTLA-4) APC,17-1529-42,Thermo Fisher Scientific (eBioscience),1/20, Clone 14D3, Lot 2297184
CD223 (LAG3) PE,130-105-452,Miltenyi Biotech,1/11, Clone REA351, Lot 5180529177
CD244 PE-Cy7,329520,Biolegend,1/80, Clone C1.7, Lot B293520
CD271 (NGFR) PE-Cy7,345110,Biolegend,1/100 , Clone ME20.4, Lot B258061
CD273 (PD-L2) APC,329608,Biolegend,1/20 , Clone 24F.10C12, Lot B258334; B364924
CD274 (PD-L1) BV421,329714,Biolegend,1/50 , Clone 29E.2A3, Lot B240740
CD278 (ICOS) APC,313510,Biolegend,1/40 , Clone C398.4A, Lot B248382
CD279 (PD-1) BV421,562516,BD Horizon,1/40 , Clone EH12.1, Lot 8345993
CD303 BV421,566427,BD Horizon,1/40 , Clone V24-785, Lot 7279714; 8058837
EOMES PE-Cy7,25-4877-42,Thermo Fisher Scientific (eBioscience),1/20 , Clone WD1928, Lot 1923396
Fc block,564220,BD,1/200 Clone Fc1, Lot 1315765
FLAG APC, Miltenyi Biotech, 130-119-683, 1/50 , Clone REA216, Lot 5220906095
Fibroblast PE, Miltenyi Biotech, 130-100-136, 1/11 , Clone REA165, Lot 5190918383

Fixable Near-IR Dead Cell Stain,L34976,Thermo Fisher Scientific,1/100
FOXP3 AlexaFluor488,320212,Biolegend,1/20 , Clone 259D, Lot B268810
FOXP3 PE-CF594,563955,BD Horizon,1/20 , Clone 236A/E7, Lot 00433311; 7235501
Granzyme B AlexaFluor700,560213,BD Pharmingen,1/100, Clone GB11, Lot 7156691
HLA-A2 PE-Cy7,561347,BD Pharmingen,1/100 , Clone BB7.2, Lot 9315578
HLA-A, B, C AlexaFluor 700,311438,Biolegend,1/80,Clone W6/32, Lot B254073; B222586
HLA-A, B, C PE,311406,Biolegend,1/100, Clone W6/32, Lot B265864
HLA-DR FITC,307604,Biolegend,1/100, Clone L243, Lot B223614
HLA-DR APC,307610,Biolegend,1/80, Clone L243, Lot B214996
HLA-DR, DP, DQ BUV395, 740302,BD OptiBuild,1/150, Clone Tu39, Lot 7347943
Human lgG4-Fc PE,9200-09,Southern Biotech,1/100, Clone HP6025, Lot B3317-YD17Z
IFNgamma AlexaFluor647,563495,BD Pharmingen,1/20, Clone 4S.B3, Lot 9151891
lsotype control,400202, Biolegend,as indicated, Lot B241031
Kl67 APC,17-5699-42,Thermo Fisher Scientific (eBioscience),1/200, Clone 20Raj1, Lot 4329350
Pembrolizumab, Merck, 20ug/ml, Clone MK-3475, Lot T003543
SOX1O AlexaFluor488,sc-365692 AF488,Santa Cruz,1/50, Clone A-2, Lot K1116
TBET BV421,563318,BD Horizon, 1/20 , Clone O4-46, Lot 7040515
TCRalphabeta APC,306718,Biolegend,1/80, Clone IP26, Lot B236612
TCRgammadelta BV421,744870, BD Optibuild,1/80, clone 11F2, Lot 7352676

Antibodies for immunoblotting
ß2-microglobulin (1:1000; D8P1H; Cell Signaling Technology; Cat No. 12851, Lot: 01)
CIITA (1:1000; Cell Signaling Technology; Cat No. 3793, Lot: 02)
STAT1 (1:1000; 9H2; Cell Signaling Technology; Cat No. 9176, Lot: 08)
phospho-STAT1 Ser727 (1:1000; D3B7; Cell Signaling Technology; Cat No. 8826, Lot: 01)
JAK1 (1:1000, Cell Signaling Technology; Cat No. 3332, Lot: 06)
JAK2 (1:1000, E4Y4D; Cell Signaling Technology; Cat No. 74987, Lot: 01),
IRF1 (1:1000; D5E4; Cell Signaling Technology; Cat No. 8478, Lot: 02),
AXL (1:200; R&D systems; Cat No. AF154, Lot: DMG0618111)
MLANA/Mart-1 (1:1000; Cell Signaling Technology; Cat No. 34511, Lot: 01)
MITF (1:1000; C5; Calbiochem; Cat No. OP126L)
SOX10 (1:1000; D5V9L; Cell Signaling Technology; Cat No. 89356, Lot: 01)
ß-Actin (1:6000; AC-74; Sigma-Aldrich; Cat No. A5316, Lot: 066M 4820V)
Secondary antibodies used were IRDye 680RD Goat anti-Mouse IgG (Cat no: 926-68070, Lot: C80926-17), IRDye 680RD Donkey anti-Goat IgG (Cat no: 926-68074, Lot: C80207-04) and IRDye 800CW Goat anti-Rabbit IgG (Cat no: 926-32211, Lot: C90220-05), all at 1:20,000 (LI-COR, Lincoln, NE) for fluorescent detection and Goat anti-Mouse Immunoglobulin HRP (Cat no: P0447, Lot: 20070514) and Goat anti-Rabbit Immunoglobulin HRP (Cat no: P0448, Lot:20042622) , all at 1:6000 (Agilent Dako, Santa Clara, CA)

Validation

The antibodies are commercially available and validated by the manufacturer. We used healthy donors peripheral blood cells and melanoma tumor dissociates to confirm the staining specificity and determine dilutions for antibodies used in flow cytometry. Cell signaling applies the work of Uhlen, et. al. ("A Proposal for Validation of Antibodies." Nature Methods (2016)) for antibody validation. Antibody specificity, signal strength are validated in various model systems and correlated with target expression in antibody independent assays.

For primary antibodies used in immunoblotting:
ß2-microglobulin (1:1000; D8P1H; Cell Signaling Technology; Cat No. 12851). According to web site (https://www.cellsignal.com/products/primary-antibodies/b2-microglobulin-d8p1h-rabbit-mab/12851) - the antibody is validated at 1:1000 dilution for WB of human ß2M, and published for WB in multiple studies (e.g. PMID:34162872)

CIITA (1:1000; Cell Signaling Technology; Cat No. 3793). According to web site (https://www.cellsignal.com/products/primary-antibodies/ciita-antibody/3793) - the antibody is validated at 1:1000 dilution for WB of human CIITA and published for WB in multiple studies (e.g. PMID:35760800)

STAT1 (1:1000; 9H2; Cell Signaling Technology; Cat No. 9176). According to web site (https://www.cellsignal.com/products/primary-antibodies/stat1-9h2-mouse-mab/9176) - the antibody is validated at 1:1000 dilution for WB of human STAT1, and published for WB in multiple studies (e.g. PMID:35840558)

phospho-STAT1 Ser727 (1:1000; D3B7; Cell Signaling Technology; Cat No. 8826). According to web site (https://www.cellsignal.com/products/primary-antibodies/phospho-stat1-ser727-d3b7-rabbit-mab/8826) - the antibody is validated at 1:1000 dilution for WB of human p-STAT1Ser727, and published for WB in multiple studies (e.g. PMID:36323660)

JAK1 (1:1000, Cell Signaling Technology; Cat No. 3332). According to web site (https://www.cellsignal.com/products/primary-antibodies/jak1-antibody/3332) - the antibody is validated at 1:1000 dilution for WB of human JAK1, and published for WB in multiple studies (e.g. PMID:35760800)

JAK2 (1:1000, E4Y4D; Cell Signaling Technology; Cat No. 74987). According to web site (https://www.cellsignal.com/products/primary-antibodies/jak2-e4y4d-mouse-mab/74987) - the antibody is validated at 1:1000 dilution for WB of human JAK2, and published for WB in multiple studies (e.g. PMID:32276266)

IRF1 (1:1000; D5E4; Cell Signaling Technology; Cat No. 8478). According to web site (https://www.cellsignal.com/products/primary-antibodies/irf-1-d5e4-xp-rabbit-mab/8478) - the antibody is validated at 1:1000 dilution for WB of human IRF1, and published for WB in multiple studies (e.g. PMID:36594555)

AXL (1:200; R&D systems; Cat No. AF154). According to web site (https://www.rndsystems.com/products/human-axl-antibody_af154) - the antibody is validated at 1ug/ml (1:200 dilution) for WB of human AXL1, and published for WB in multiple studies (e.g. PMID:35927581)

MLANA/Mart-1 (1:1000; Cell Signaling Technology; Cat No. 34511). According to web site (https://www.cellsignal.com/products/primary-antibodies/mlana-mart-1-antibody/34511) - the antibody is validated at 1:1000 dilution for WB of human MLANA, and was used by us previously (e.g. PMID:32312968)

MITF (1:1000; C5; Calbiochem; Cat No. OP126L). According to the datasheet, the antibody was tested using B16 cells or skin melanoma tissue as positive control, and was used by us previously (PMID: 34073253), and in multiple studies (e.g. 29484133)

SOX10 (1:1000; D5V9L; Cell Signaling Technology; Cat No. 89356). According to web site (https://www.cellsignal.com/products/primary-antibodies/sox10-d5v9l-rabbit-mab/89356) - the antibody is validated at 1:1000 dilution for WB of human SOX10, and published for WB in multiple studies (e.g. PMID:36437876)

ß-Actin (1:6000; AC-74; Sigma-Aldrich; Cat No. A5316). According to web site (https://www.sigmaaldrich.com/AU/en/product/sigma/a5316) - the antibody is validated at 1:5000 dilution for WB of human ß-actin, and published for WB in multiple studies (e.g. PMID:14660748)

Eukaryotic cell lines

Policy information about cell lines and Sex and Gender in Research

Cell line source(s)	Short term melanoma cell lines including SCC16-0016 were generated from patient biopsies as detailed in Methods. Details of the patients including sex are provided in Table 1 and details of the cell lines are provided in Tables S1 and S2.
Authentication	Baseline profiling was performed on each cell at the earliest possible passage using Cell authentication of established melanoma cell lines and confirmed using the StemElite ID system from Promega.
Mycoplasma contamination	All cells tested negative for mycoplasma using the MycoAlert Mycoplasma Detection Kit from Lonza
Commonly misidentified lines (See ICLAC register)	No commonly misidentified cell lines were used in this study

Clinical data

Policy information about clinical studies

All manuscripts should comply with the ICMJEguidelines for publication of clinical research and a completedCONSORT checklist must be included with all submissions.

Clinical trial registration	This study was a retrospective study of melanoma patient samples collected according to the TEAM protocol at MIA, and was not part of a clinical trial.
Study protocol	X15-0454
Data collection	This study included metastatic melanoma patients who were treated with PD-1 inhibitors (pembrolizumab or nivolumab) at Melanoma Institute Australia (MIA) and affiliated hospitals.
Outcomes	Patient demographics and clinicopathologic features including Eastern Cooperative Oncology Group (ECOG) performance status, lactate dehydrogenase (LDH) levels at baseline, BRAF and NRAS mutation status, American Joint Committee on Cancer (AJCC) stage 8th Edition and disease distribution were collected. Investigator-determined objective response was assessed radiologically with computed tomography (CT) scans alone or where indicated, with magnetic resonance imaging (MRI) of the brain, at two to three monthly intervals using irRECIST.

Flow Cytometry

Plots

Confirm that:

- [x] The axis labels state the marker and fluorochrome used (e.g. CD4-FITC).
- [x] The axis scales are clearly visible. Include numbers along axes only for bottom left plot of group (a 'group' is an analysis of identical markers).
- [x] All plots are contour plots with outliers or pseudocolor plots.
- [x] A numerical value for number of cells or percentage (with statistics) is provided.

Methodology

Sample preparation	Tumour biopsies were enzymatically processed and dissociated into single cell suspensions using the Tumor Dissociation Kit and gentleMACS Dissociator (Miltenyi Biotec). Single cell suspensions were frozen as tumour dissociates in 10% DMSO in human serum. Tumour dissociates were thawed in TIL medium containing 20ug/ml DNAse type II-S and washed prior to flow cytometry staining.
Instrument	5-laser BD LSRFortessa X20 flow cytometer from BD Biosciences
Software	FlowJo v10 software from BD Biosciences
Cell population abundance	HLA-A2 transfected WMD17-0112 cells were stained and flow-sorted for HLA-A2high fraction 24 post transfection, using MACSQuant Tyto (Miltenyi Biotech). Sorted cells were >97% HLA-A2high, as determined by flow cytometry. HLA-A2 expression on PRE-sorted and untransfected WMD17-0112 cells was also assessed.
Gating strategy	We applied FSC and SSC parameter to exclude cell debris and doublets and live/dead staining to select live cells.<br>Melanoma was identified as CD45-neg_SCC-A-int to high_Fibroblast-neg_SOX-10+<br>Tumour infiltrating lymphocytes identified as CD45+_SCC-A-low_CD3+<br>Granulocytes/myeloid-derived suppressor cells as CD45-int_SCC-A-int_CD15+<br>Tumor-associated macrophages as CD45+_SCC-A-high_CD3-neg-CD19-neg_HLA-DR+_CD64+<br>Monocytes as CD45+_SCC-A-int_CD3-neg-CD19-neg_HLA-DR+_CD64-int<br>Plasmacytoid dendritic cells as CD45+_SCC-A-low_CD3-neg-CD19-neg_HLA-DR+_CD64-neg_CD303+<br>CD141+ dendritic cells as CD45+_SCC-A-low_CD3-neg-CD19-neg_HLA-DR+_CD64-neg_CD303-neg_CD1c-neg_CD1<br>CD1c+ dendritic cells as CD45+_SCC-A-low_CD3-neg-CD19-neg_HLA-DR+_CD64-int_CD303-neg_CD141-neg_CD1<br>T cells, TCR alpha beta as CD45+_SCC-A-low_CD3+_TCRab+ .<br>T cells, TCR gamma delta as CD45+_SCC-A-low_CD3+_TCRgd+<br>CD8 T cells as CD45+_SCC-A-low_CD3+_TCRab+_CD4-neg_CD8+<br>Conventional CD4 T cells (Tconv) as CD45+_SCC-A-low_CD3+_TCRab+_CD8-neg_CD4+_FOXP3-neg<br>B cells as CD45+_SCC-A-low_CD3-neg_CD19+<br>NK cells as CD45+_SCC-A-low_CD3-neg_CD244+_CD56+/int<br>Gating strategy is described in Supplementary Information (Table S2)

☒ Tick this box to confirm that a figure exemplifying the gating strategy is provided in the Supplementary Information.
