# Supplementary material for: The molecular and functional landscape of resistance to immune checkpoint blockade in melanoma
Source: Nat Commun. 2023 Mar 18;14:1516. doi: 10.1038/s41467-023-36979-y (PMC10024679; doi:10.1038/s41467-023-36979-y)
Supplement: Supplementary file 4 — Description of Additional Supplementary Files [file 41467_2023_36979_MOESM4_ESM.docx]

**Description of Additional Supplementary Files**

Supplementary Data 1

Description: Gene-sets differentially expressed (q<0.05) in PD1 PROG cells with high baseline (n=6) vs low baseline (n=15) IFNγ signaling

Supplementary Data 2

Description: Genes differentially expressed (q<0.05) in PD1 PROG cells with high baseline IFNγ signaling (n=6) vs low baseline IFNγ signaling (n=15)

Supplementary Data 3

Description: Secreted cytokines differentially expressed in PD1 PROG cells with high baseline IFNγ signaling (n=6) vs low baseline IFNγ signaling (n=15)

Supplementary Data 4

Description: Secreted cytokines differentially expressed in 21 PD1 PROG cells treated with vehicle control vs 1000U/ml IFNγ for 72h

Supplementary Data 5

Description: Sanger sequence data of CIITA pIV promoter derived from the PD1 PROG cell lines SMU17-0263 and SCC11-0270

Supplementary Data 6

Description: HLA-Typing of PD1 PROG and PRE melanoma cells and matched germline DNA
